# Bio-based products control black rot (*Xanthomonas campestris* pv. *campestris*) and increase the nutraceutical and antioxidant components in kale

**DOI:** 10.1038/s41598-018-28086-6

**Published:** 2018-07-05

**Authors:** Andrés M. P. Nuñez, Gabriel A. A. Rodríguez, Fernando P. Monteiro, Amanda F. Faria, Julio C. P. Silva, Ana C. A. Monteiro, Carolina V. Carvalho, Luiz A. A. Gomes, Ricardo M. Souza, Jorge T. de Souza, Flávio H. V. Medeiros

**Affiliations:** 1 Department of Plant Pathology, Universidade Federal de Lavras, 37200-000 Lavras, Minas Gerais Brazil; 2 Department of Nutrition, Universidade, Federal de Lavras, 37200-000 Lavras, Minas Gerais Brazil; 3 Department of Agriculture, Universidade Federal de Lavras, 37200-000 Lavras, Minas Gerais Brazil; 4 Present Address: Universidad de San Carlos, Avenida 11, Guatemala, 01012 Guatemala

## Abstract

Black rot of crucifers, (*Xanthomonas campestris* pv. *campestris*) is the principal yield-limiting and destructive pathogen of cruciferous crop worldwide. In order to validate a bio-based control alternative for this disease, whey, lime sulfur, biofertilizer, Bordeaux mixture or raw milk were applied to kale (*Brassica oleracea* var. *acephala*) plants. The disease control was achieved by most of the tested products. Milk-based products (raw milk and whey) and biofertilizer reduced the severity by 44 and 56% in the field. Antioxidants, crude fibber, crude protein and lipid contents and kale yield were verified in the five treatments on the leaves with and without *X. campestris* pv. *campestris* inoculation. In the absence of the pathogen (non-inoculated), lime sulfur and Bordeaux mixture improved plant nutritional value compared to organic treatments, nevertheless milk-based products and biofertilizer improved the evaluated variables more than the control. However, on leaves inoculated with *X. campestris* pv. *campestris* raw milk increased antioxidant activity, crude protein and fiber contents, whereas biofertilizer increased kale yield, lipid and antioxidant contents. Milk-based products and biofertilizer were further evaluated in greenhouse trials to determinate the activity of defense-related enzymes and lignin content. Biofertilizer treatment resulted in increased phenylalanine ammonia lyase, catalase, peroxidase activities and lignin content. Hence, the application of milk-based products and biofertilizer are promising to control black rot of crucifers and also improves food quality by boosting nutritional values and antioxidant activity.

## Introduction

The Cruciferae or Brassicaceae family is composed of approximately of 338 genera and 3,709 species worldwide^1^, where the *Brassica* group has the greatest socio-economic impact throughout the popular vegetable crops for human food, fodder, oil seed crops, antioxidant-based diet, nutraceutical compounds for cancer, biofuels and biofumigants^2–4^. The main brassica vegetable crop cultivated is *Brassica oleracea*, which includes the varieties *B. oleraceae* var. *capitata* (cabbage), *B. oleraceae* var. *botrytis* (cauliflower), *B. oleraceae* var. *italic* (broccolis), *B. oleraceae* var. *gemmifera* (Brussels sprouts), and *B. oleraceae* var. *acephala* (kale)^5,6^. Among the Brassicaceae, kale is a popular vegetable, largely consumed in tropical areas and mostly produced by small growers and urban gardeners. Its consumption is particularly attractive due to its nutritional and antioxidant values. However, many plant diseases contribute to the quantitative and qualitative reduction in the kale production and decrease the plant yield and nutritional value^7^.

The main yield-limiting and destructive pathogen of cruciferous crop worldwide is *Xanthomonas campestris* pv. *campestris* that causes black rot. The bacterium is worldwide distributed wherever rainfall or heavy dews are plentiful with an average temperature between 25 to 30 °C^8,9^. Mustard, collards, rutabaga, turnip, cabbage, broccoli, cauliflower, Brussels sprout, radish and kale are all affected by black rot^10^. The disease is particularly devastating in cabbage and kale, where leaves are the commercial product and renders them unmarketable^8^. *X. campestris* pv. *campestris* is a constant concern for small cruciferous growers, especially when the environmental conditions are favorable and the same area is continuously cropped with the host plant^11^.

Black rot can be controlled by the use of cultural, chemical, biological control and host resistance methods^8^. Successful alternatives to control diseases in Brassicaceae with organic amendments are usually carried out in the framework of integrated pest management programs^12^. Urban and organic farmers use mainly alternative methods to control *Brassica* spp. diseases such as agro-waste-based products. Plant manure, soaks or slurries, biofertilizers and dairy products have been proposed for plant protection^8,13,14^ but not yet evaluated for the management of a bacterial disease or on kale.

Despite agro-waste-based products efficiency for the management of fungal diseases little is available for black rot management. Furthermore, studies concerning the effect of *X. campestris* pv. *campestris* and those products on the nutritional values of kale and how such products exert disease control have not been undertaken. The aim of the present work was to evaluate the efficacy of these products for the alternative control of black rot and activation of defense-related responses against the disease and evaluate variables related to nutraceutical value of kale.

## Results

### Screening of different alternative controls

**Figure 1:**
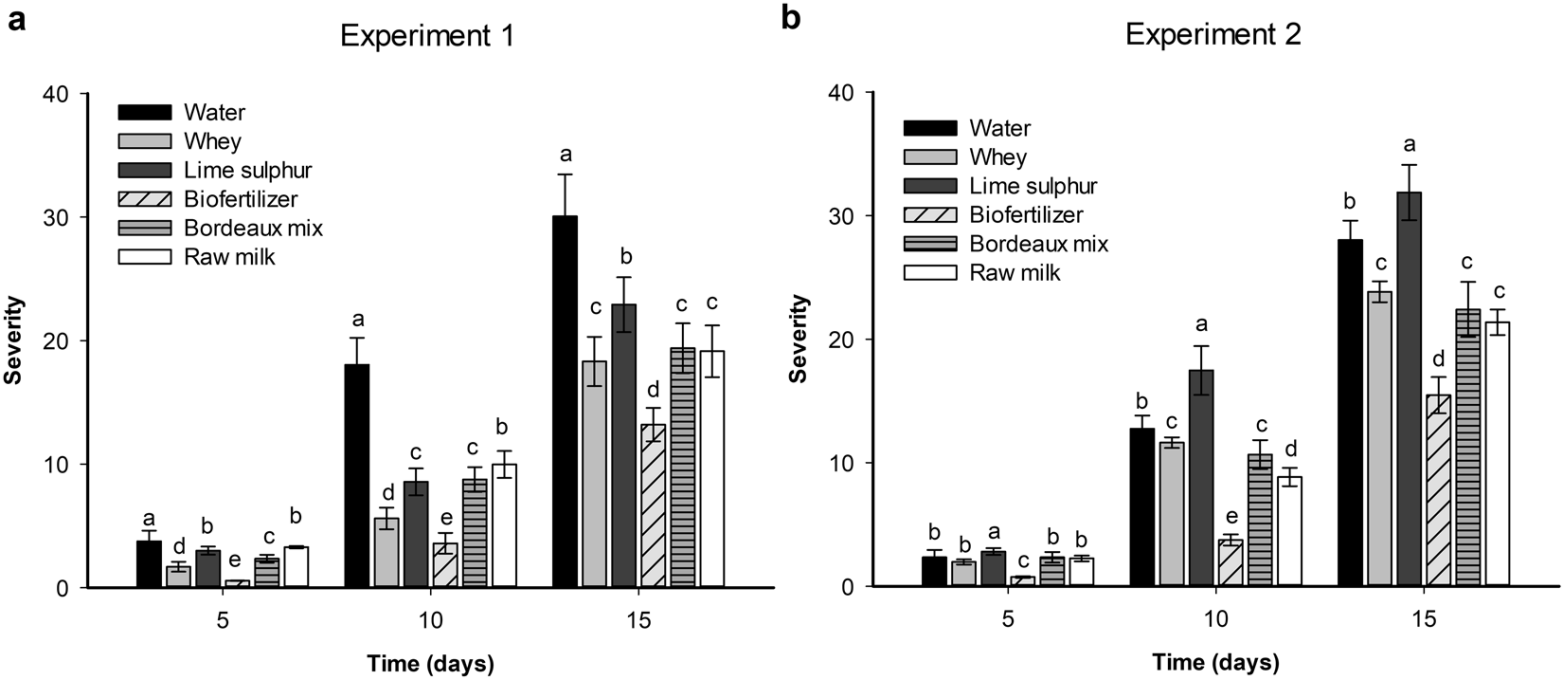
Efficacy of different treatments on the reduction of black rot severity in kale plants. The artificial inoculation of the pathogen *X. campestris* pv*. campestris* was applied eight days later than the first application of the treatments and disease severity was estimated by the area under the disease progress curve (AUDPC) in the (**a**) first and (**b**) second experiment. Mean values followed by the same letter are similar according to Tukey’s test at 5% probability. Bars represent the standard error of the mean.

Biofertilizer, whey, raw milk and Bordeaux mixture reduced disease severity as compared to the control in the evaluated periods and the efficacy was dependent on the biological replicate (experiment). For the first experiment, the reduction by biofertilizer was 85% at the fifth day after inoculation (P < 0.01), 80% at the tenth day (P < 0.01) and 56% at the fifteenth day (P < 0.01). For the second experiment, the biofertilizer reduction was 68% on the fifth day (P < 0.01), 70% on the tenth day (P < 0.01) and 44% on the fifteenth day (P = 0.05) (Fig. 1a,b). For both experiments, the treatments biofertilizer and milk-based products provided the highest reduction in the assessments. In relation to the positive controls, the Bordeaux mixture provided protection against black rot on both assessments, while lime sulfur and raw milk had the lowest reduction in the first experiment (Fig. 1a) and lime sulfur even increased black rot severity in the second experiment (Fig. 1b).

### Foliar mineral nutrients according to different treatments and inoculation

**Figure 2:**
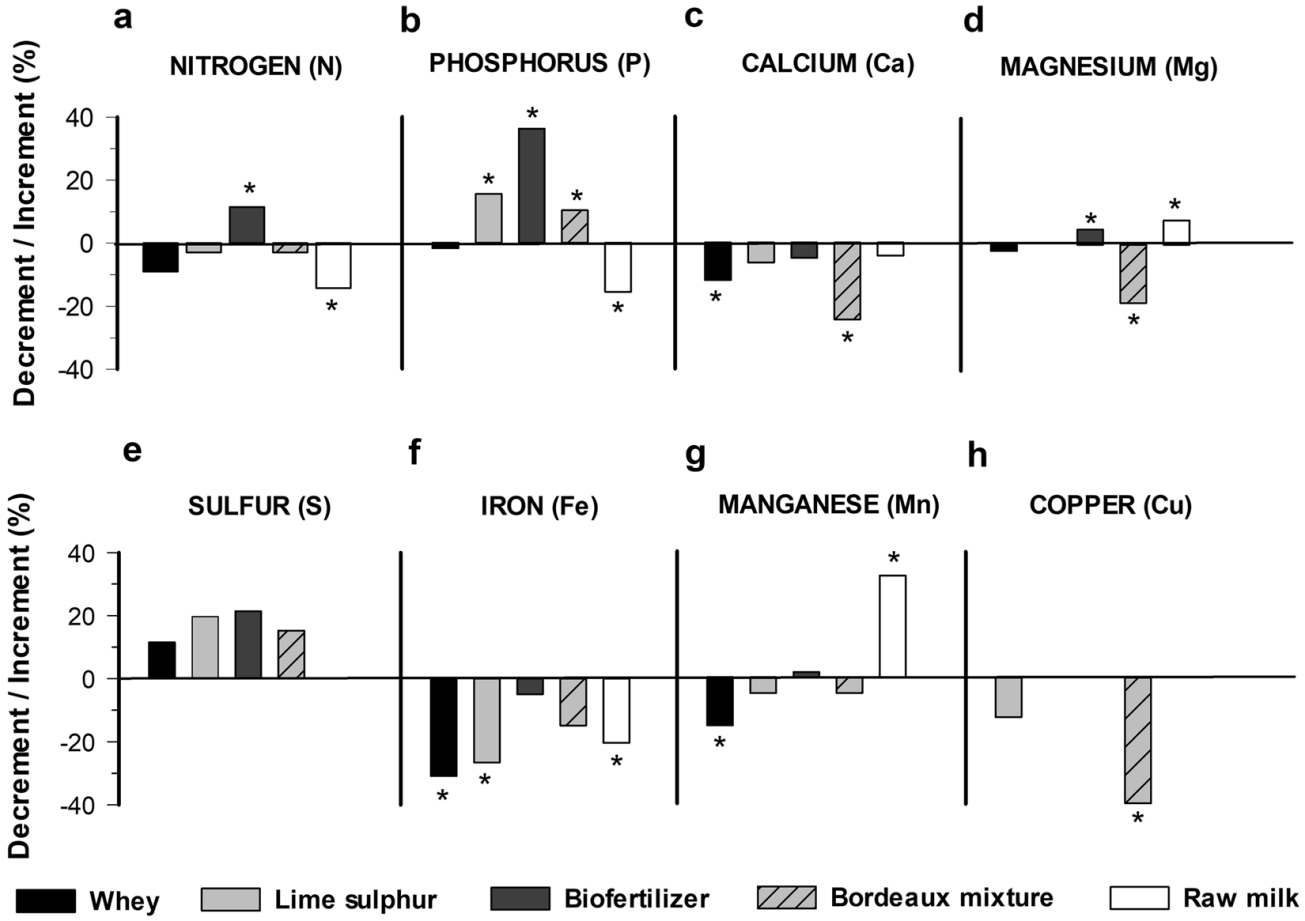
Contribution of bio-based products on the foliar mineral nutrition of kale (*Brassica oleraceae* var. *acephala*) at 23 days after transplanting. Asterisk refer to significant increment or decrement of the means compared to control mean (water) according to the Tukey test at 5% probability (original means are presented in the supplementary data Table S1).

**Figure 3:**
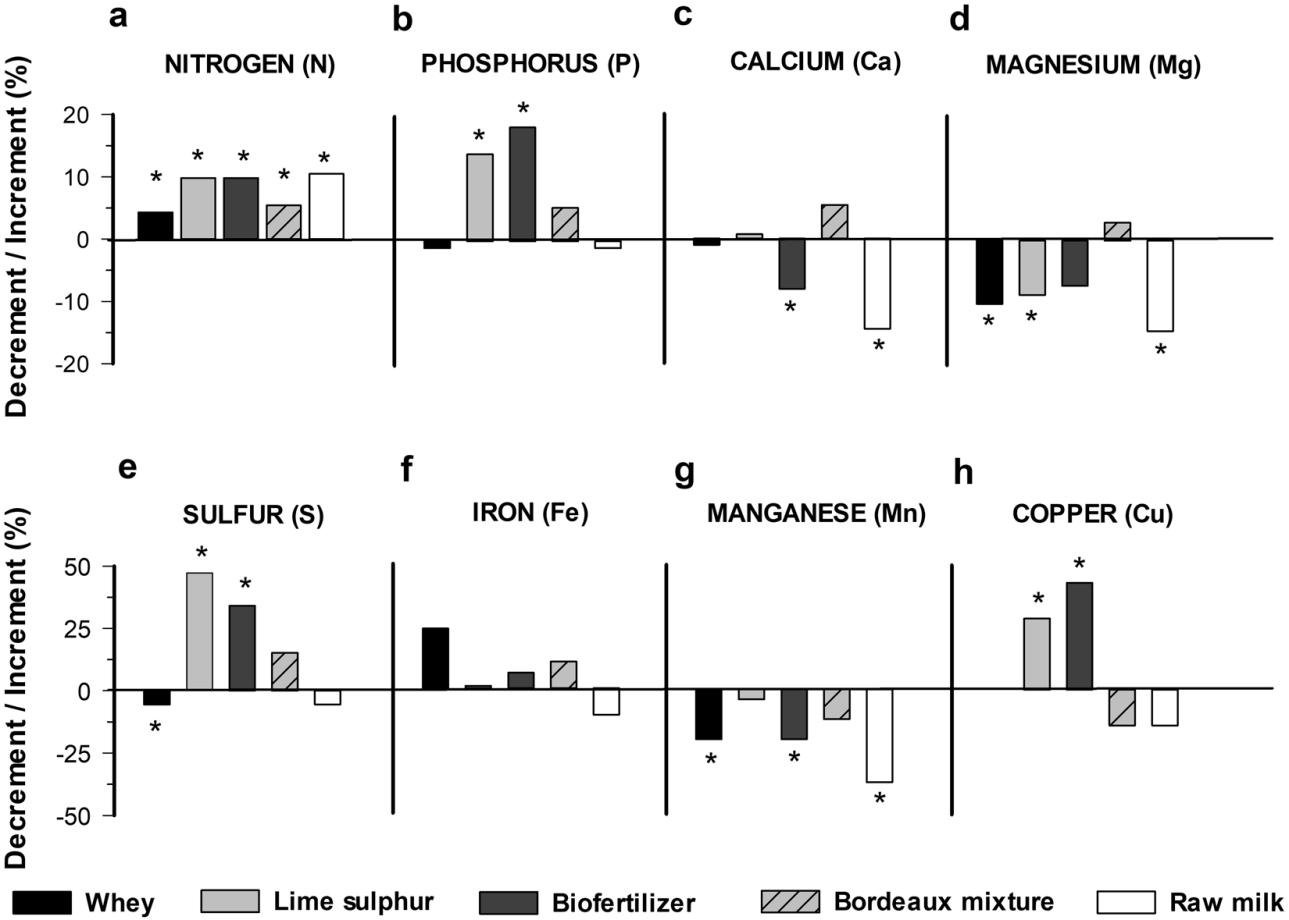
Contribution of different products on the foliar mineral nutrition of kale (*Brassica oleraceae* var. acephala) at 23 days after transplanting and eight days after inoculation with *Xanthomonas campestris* pv. *campestris*. Asterisks refer to significant increment or decrement of the means compared to the control (water) according to the Tukey test at 5% probability (original means are presented in the supplementary Table S2).

Both the inoculation and treatments contributed to a significant effect on kale foliar nutrition (Figs 2, 3; Supplementary Tables S1, S2). However, significant increases for nutrients in most treatments were observed for the inoculated experiments. This may be an artifact of the buildup and solubilization of nutrients as the experiments were planted in the same spot and the non-inoculated treatments were planted before the inoculated ones, with two biological replicates each (experiments) and, on every plant transplanted the same rate of fertilization was applied. Nevertheless, raw milk promoted an increase in nitrogen content only in plants infected with *X. campestris* pv. *campestris* (Fig. 3a), while a reduction in this nutrient was observed in the absence of infection (Fig. 2a). This finding corroborates with the significant correlation of nitrogen levels with lower black rot severity (Supplementary Table S1). A similar significant negative correlation was observed between the disease severity and phosphorus content and both lime sulfur and biofertilizer promoted an increase in the contents of this nutrient. Raw milk treatment decreased the foliar content of nitrogen, phosphorus and iron mainly for the non-inoculated (Fig. 2) and calcium and magnesium for the inoculated one (Fig. 3), i.e., the pattern of detrimental effect of milk on the foliar nutrition was dependent on the bacterial infection. Each nutrient level was evaluated for its correlation with the black rot severity in the last evaluation period (15 days). There was a significant negative correlation between nutrients and the disease severity when nitrogen (N) and potassium (P) were evaluated. This represents a decrease in severity with higher N and P treatments (Supplementary Table S3).

### Leaf yield and centesimal composition (nutraceutical)

**Figure 4:**
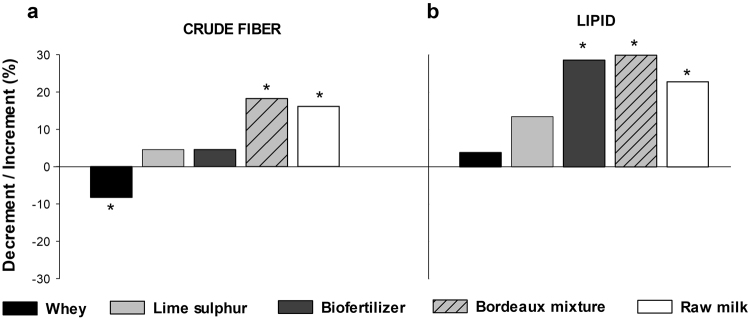
Kale (*Brassica oleraceae* var. *acephala* cv. Manteiga) (**a**) crude fiber and (**b**) lipid content with and without *Xanthomonas campestris* pv. *campestris* inoculation at 23 days after transplanting or eight days after inoculation. Asterisk refer to significant increment or decrement of the means compared to control mean (water) according to the Tukey test at 5% probability (original means are presented in the supplementary data Table S4). Maxixum F was obtained with Hartley test before running joint analyses.

**Figure 5:**
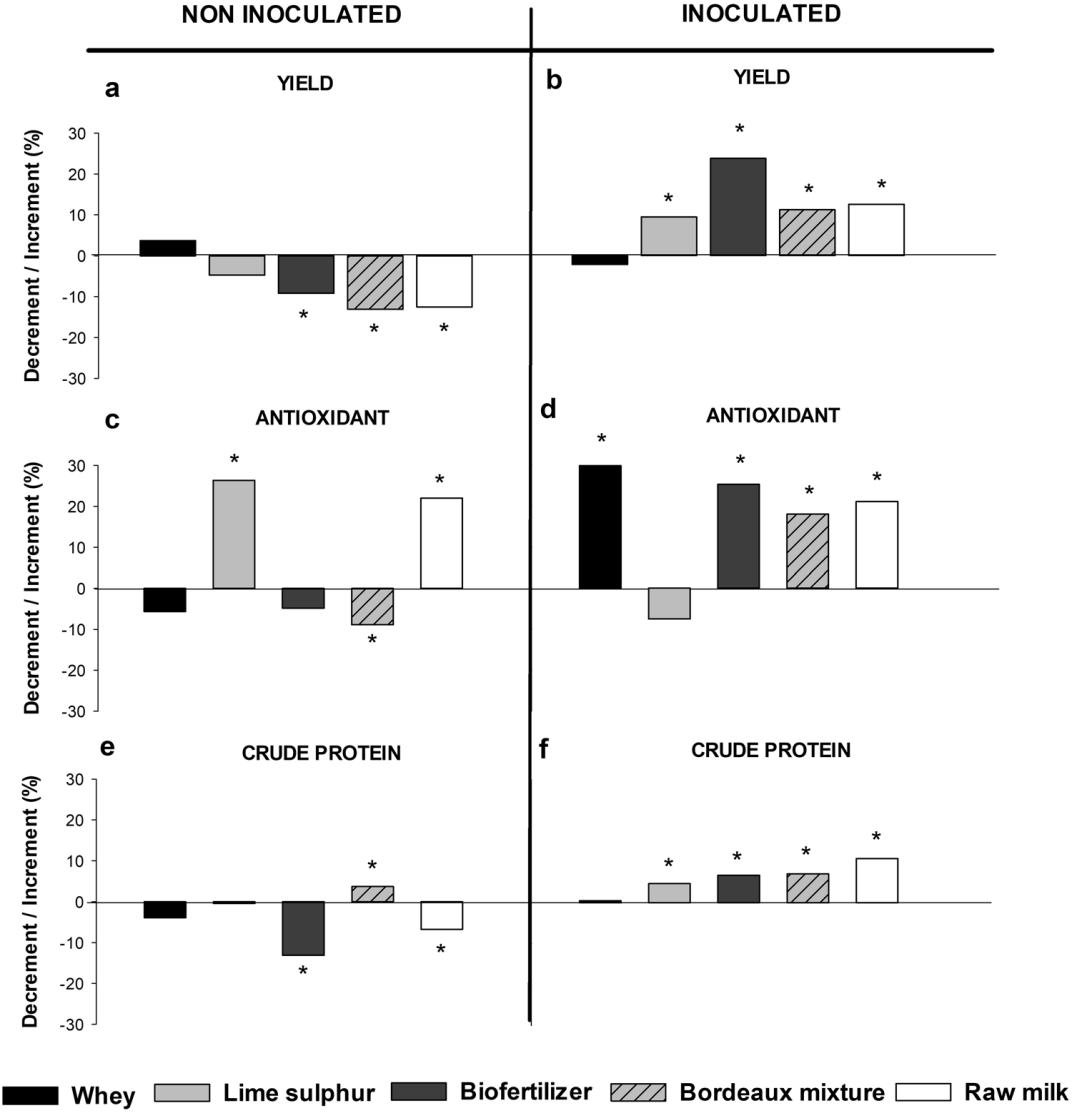
Variation of (**a**,**b**) yield, (**c**,**d**) antioxidant and (**e**,**f**) crude protein compared to water control, with or without *Xanthomonas campestris* pv. *campestris* inoculation at 23 days after transplanting or eight days after inoculation of kale (*Brassica oleraceae* var. *acephala* cv. Manteiga). Asterisk refer to significant increment or decrement of the means compared to control mean (water) according to the Tukey test at 5% probability (original means are presented in the supplementary data Table S4).

For most variables evaluated in our experiments, the pathogen inoculation had a significant impact on the plant performance and the products that assured the disease control also contributed to the improvement of plant yield and to increase the nutraceutical-related variables (Figs 4 and 5). Whenever the experiments were evaluated through Hartley’s index, the only crude fiber (Fmax = 2.014) and lipid content (Fmax = 3.69) had homoscedasticity that allowed to pool data from all four experiments together, i.e., there was no impact of the inoculation on the performance of the product (Fig. 4). For crude fiber (P < 0.01) (Fig. 4a), raw milk or Bordeaux mixture treatments resulted in the highest increment (ca. 15%) and whey treatment decreased the leaf protein content compared to the water control. In regard to lipid content (P = 0.015) (Fig. 4b), Bordeaux mixture, biofertilizer and raw milk resulted in higher increment than control with an overall similar effect on inoculated and healthy plants (Supplementary Table S4). For all other variables (Fig. 5), Hartley’s test did not show homoscedasticity of variance (Yield-Fmax: 50.40; Antioxidant-Fmax: 12,34; Crude protein-Fmax: 8.54) and therefore experiments were analyzed separately, i.e. as inoculated and non-inoculated. In relation to the assays without inoculation, control treatment had a higher yield when compared to biofertilizer, Bordeaux mixture and raw milk (P < 0.01) (Fig. 5a). Furthermore, antioxidant activity was higher for lime-sulphur and raw milk (Fig. 5c). On the other hand, Bordeaux mixture resulted in higher crude protein (P < 0.01) (Fig. 5e) in Bordeaux mixture and raw milk than all other treatments (P < 0.01). When considered the experiments with the bacterial inoculation, a higher leaf yield was obtained for biofertilizer followed by raw milk, Bordeaux mixture and lime sulphur (P < 0.01) (Fig. 5b, Suplementary Table S4). Similarly, the antioxidant activity was higher than the control and lime sulphur for all other treatments (P < 0.01) (Fig. 5d) and the crude protein had no increment only by whey application (P < 0.01) when compared with control (Fig. 5f).

### Defense-related enzymes

**Figure 6:**
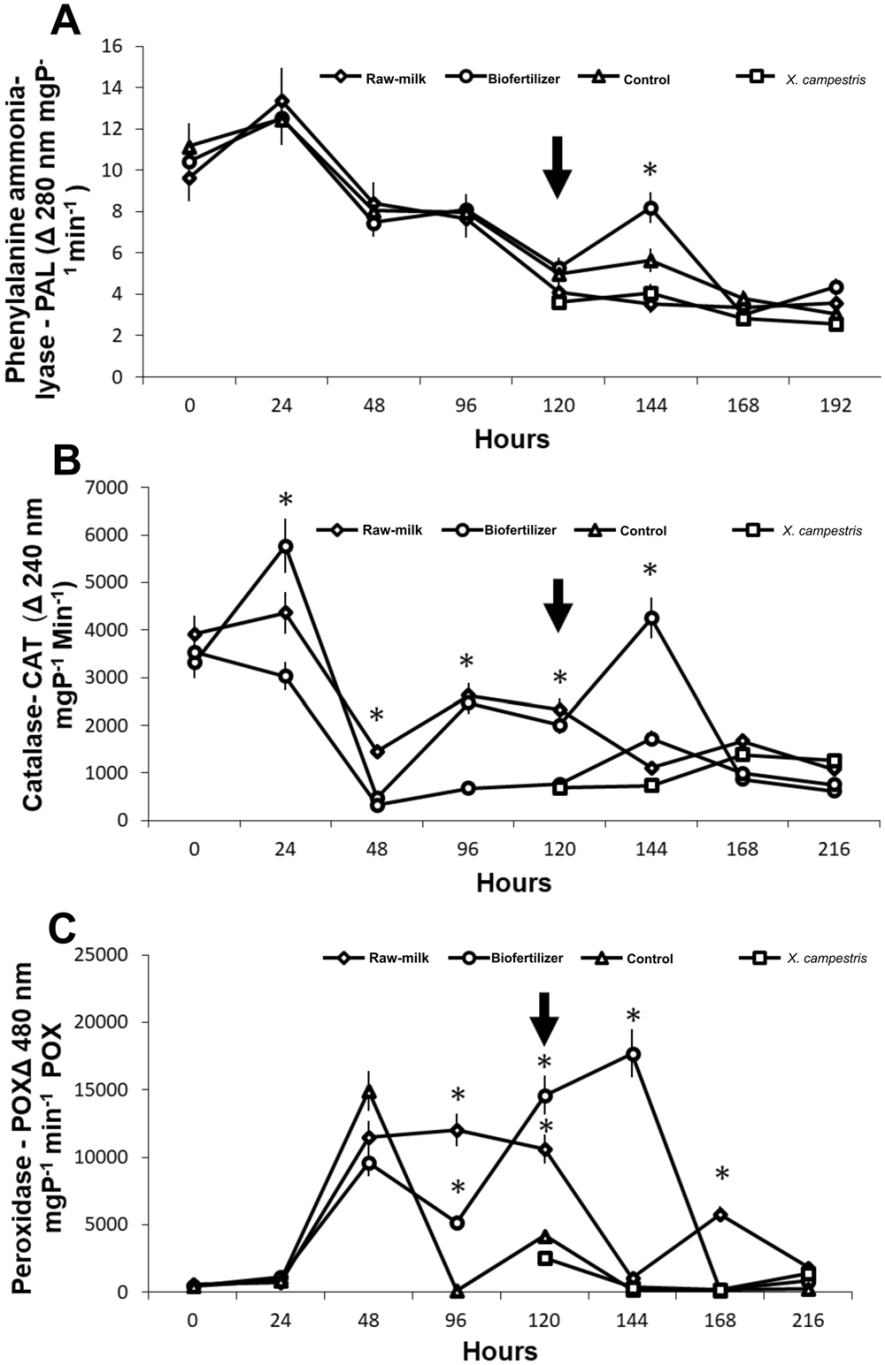
Activity of the defense-realted enzymes: (**A**) Phenylalanine ammonia-lyase, (**B**) Catalase and (**C**) Peroxidase guaiacol. Leaves of treated and control kale plants were collected and enzyme activity was determined spectrophotometrically. Black arrows indicate *X. campestris* pv. *campestris* inoculation. Asterisk indicate means that are significantly different among treatments according to the Tukey test at 5% probability.

The enzyme phenylalanine ammonia-lyase (PAL) was not induced by the treatments before inoculation, while biofertilizer induced PAL activity in kale challenged with *X. campestris pv. campestris* at 144 h, 24 h after the pathogen inoculation (Fig. 6A). The activity of the antioxidant enzyme catalase (CAT) was induced by the treatments alone till 144 h and declined thereafter in all treatments, with a higher activity of CAT for plants treated with biofertilizer 24 hours after inoculation (Fig. 6B). The peroxidase (POX) induction was high under biofertilizer treatment at 120 and 144 h, i.e, at upon inoculation and 24 hours later. However, 48 hours after inoculation the whey showed a higher POX activity than biofertilizer (Fig. 6C).

### Total phenol content and lignin quantification

**Figure 7:**
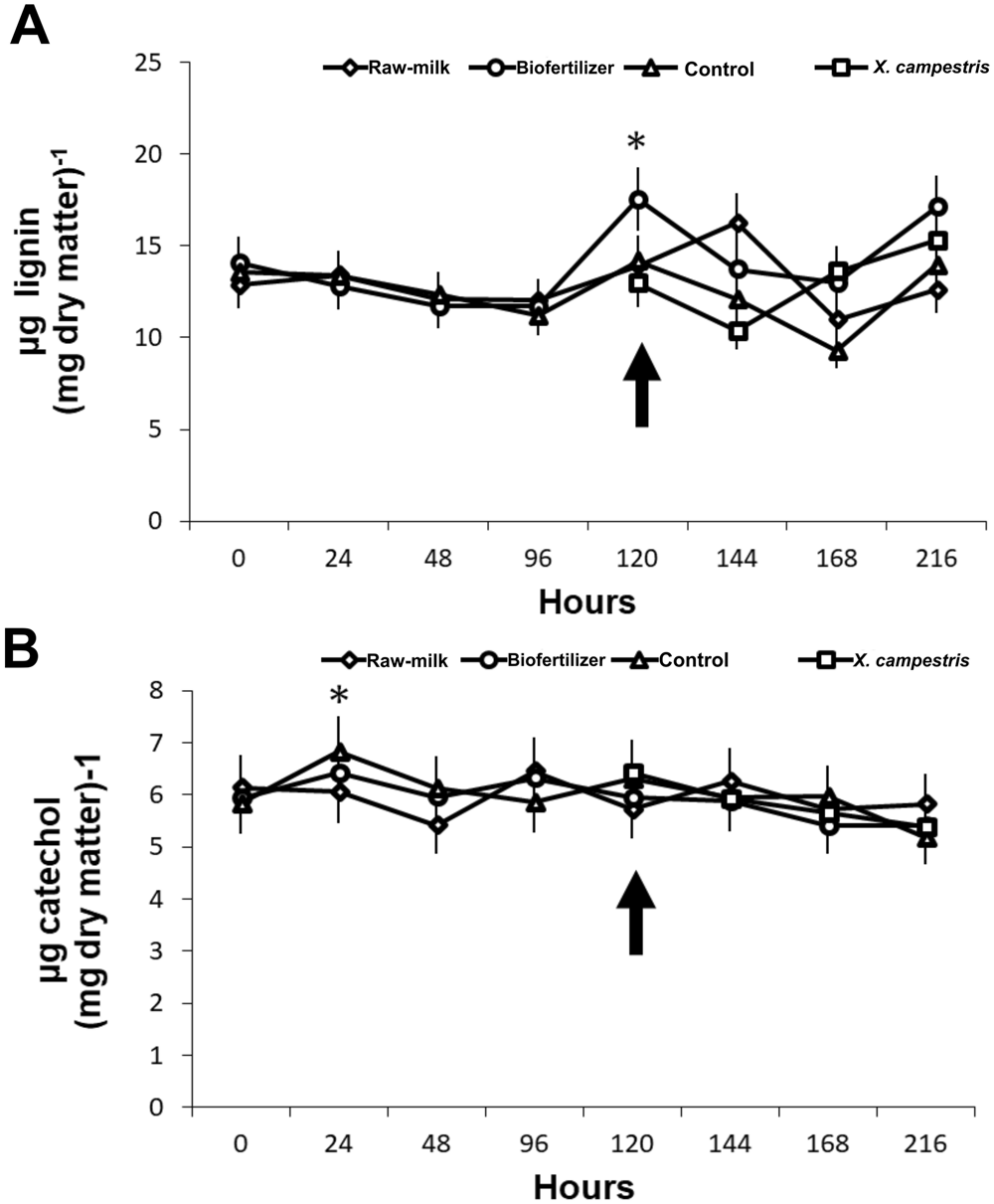
Concentration of (**A**) total phenol and (**B**) lignin in kale leaves treated with Raw milk, Biofertilizer, Control (Water), and *X. campestris pv. campestris* (*Xanthomonas campestris* pv. *campestris*. Total phenol and lignin were extracted from leaves and quantified spectrophotometrically. Black arrows indicate *X. campestris* pv. *campestris* inoculation. Asterisks indicates means that are significantly different among treatments according to the Tukey test at 5% probability.

The lignin contents in kale plants, at 120 hours (the moment of the inoculation with the pathogen), increased in plants treated with biofertilizer (P < 0.01) (Fig. 7A). The levels of phenol were not significantly different among the treatments (P = 0.08) (Fig. 7B).

## Discussion

Alternative low-cost products are effective in controlling black rot and may contribute to the increase in the nutraceutical value of kale even in the absence of the disease i.e., a preventive-basis recommendation would result in benefits beyond plant protection for the grower and consumer.

Although most tested products exert plant protection benefits, the biofertilizer promoted higher protection compared to the other tested products (Fig. 1). Biofertilizers have been studied for plant protection for years, either by the direct effect of their living micro-organisms or by the metabolites produced by them and, moreover, by activating defense mechanisms in plants tissues^15–17^. Biofertilizers are based on plant and/or animal manures and encompass an antagonistic microbial community^18,19^. For instance, tomato wilt suppressive bacteria *Chryseomonas luteola, Serratia liquifaciens* and *Aeromonas hydrophila* have already been isolated from animal manures^20^ and implicated in the plant protection benefit exerted by biofertilizers^21,22^. Furthermore, the N and P richness correlated negatively with *X. campestris pv*. *campestris* severity. Biofertilizer also contains nutrients and high availability of N and P and, although the biofertilizer composition was not analysed from nutrient content (Figs 2 and 3), the biofertilizer application assured an increase for those nutrients to the plants which in turn ensures plant immunity^23^ that result in low black rot severity.

Furthermore, when biofertilizer was sprayed onto plants, it exerted a priming-like benefit, i.e., the activity of defense-related enzymes peroxidase, catalase and phenylalanine ammonia lyase increase after inoculation. The priming activity is a key feature of resistance inducers that result in the accumulation of proteins that act as transcription factors and/or with signal transduction activity are rapidly activate genes once the plant encounters pathogen elicitors^24^.

The milk-based treatments (whey and raw milk) also reduced the black rot severity and to the best of our knowledge, this is the first report of the potential of milk-based product on a bacterial disease protection, despite its already proven efficacy to fungal and virus diseases^23,25^. Milk is composed of nutrients and microorganisms that act in synergy to promote plant protection^14^ and some of its fractions such as lactoferrin have antimicrobial activity and is part of the whey fraction^26^, i.e. is likely found as part of both tested milk-based products.

On the other hand, Bordeaux and sulfur mixture demonstrated less protection against *X. campestris pv. campestris*, therewith lime sulfur even increased the disease severity. The experiments were carried out during the rainy and warmer season (October–December), which is more conducive to black rot. The rain may also have contributed to the reduced performance of the plant protection exerted by the tested inorganic mixtures. By washing them off the leaves the duration of plant protection is reduced^27^ and the bacterial pathogen gains access to the plants. Furthermore, the observed severity increase caused by Lime sulfur may be related to low activity against the bacterial pathogen along with a high activity against non-target beneficial microorganisms growing on leave surface, creating a biological vacuum effect that facilitates the pathogen multiplication^28^. This product has phytotoxic effects on plants^29^ and, although not visually detected on kale in our experiments, the phytotoxicity may have impaired photosynthesis and resulted in reduced plant production.

Regardless bacterial inoculation, plant crude fiber increased by applying Bordeaux mixture and raw milk, whereas lipid content increased by applying biofertilizer, Bordeaux mixture and raw milk (Fig. 4). However, plant yield was similar to all treatments. Without bacterial inoculation the treatments of lime sulfur, biofertilizer and Bordeaux mixture resulted in higher levels of foliar nutrition of nitrogen and phosphorus (Fig. 2), whereas, under the same conditions, lime sulphur, Bordeaux mixture and raw milk treatments increased antioxidant and crude protein, i.e. nutraceutical-related variables (Fig. 5). However, in bacterial inoculated plants, we obtained an increase in foliar nitrogen content (Fig. 3), kale yield and high levels of the nutraceutical variables (Fig. 5) under inorganic treatments, biofertilizer and milk-based products. Moreover, the correlation of phenol content and antioxidant activity were positively and significantly correlated only when *X. campestris pv. campestris* was inoculated which implies on the boost up of such arsenal of the antimicrobial and scavenging system upon infection.

By applying the bio-based products on the healthy leaf surface, a possible priming effect was identified in the greenhouse experiment as discussed above, which would imply in the reduction in the fitness cost and consecutively absence of negative effect on plant yield^30^. This was not the case in the non-inoculated field trial, where a lower productivity was identified (Fig. 5a). Such negative effect may be overcome by a reduction in the biofertilizer concentration or the product application towards the stem as a drench directed to the leaves, which likely result in a smaller absorption of the product by the plant and therefore a milder triggering of the defensive secondary metabolism in a priming-like state in the absence of the pathogen.

Noteworthy, the commercialization of kale is not based on its weight but on the individual leaf or a set of leaves that are pulled together to make a single product and its quality is more important than its weight^31^. In this regard, the bio-based products identified as most promising for the disease control (Fig. 1) would definitively benefit the grower in the commercialization of their kale production and would even save him on transportation costs with a lighter production.

Furthermore, the increase in plant nutraceutical-related variables and yield in *X. campestris pv. campestris* infected kale denotes for the role of the products in the plant protection that may be related to stimulation of the antagonistic microbiota on the leaves surface that results in plant growth promotion and health^32,33^. Moreover, higher levels of phenols, amino acids, carbohydrates and proteins may indicate a defense mechanism activation, as it has already been shown to *Brassica* spp. against pathogenic bacteria^34^. In addition, higher levels of phenols, lipids, proteins and fibers benefit the human health and stimulate kale alternative methods of controlling diseases as the milk-based products and biofertilizer. Higher phenols and antioxidant activity in kale after milk-based products and biofertilizer application may increase proprieties of kale leaves of scavenging free radicals in the human body^35,36^.

Biofertilizer application has shown the higher enzymatic activity of POX, CAT and other defense mechanism activities after bacterial inoculation when compared to the other treatments. Such defense activation may be related to the richness of nutrients, minerals and microorganisms of the product acting as resistance inducers^37^. POX and CAT act as direct inhibitors of reactive oxygen species (ROS), specifically hydrogen peroxide (H_2_O_2_) produced by superoxide dismutase (SOD). The ROS-mediated defense responses include phytoalexin formation, which inhibits the growth of plant pathogens^38^. Thus, the *X. campestris pv. campestris* inoculation could stress the leaves blocking the electron transport chain originating OH^−^ e O_2_^−^ that are scavenged by defense enzymes increased by biofertilizer. Furthermore, biofertilizer application can activate many defense mechanisms by non-pathogenic microorganisms growth on the surface or even abiotic compounds acting as effectors, e.g., the hypersensitive reaction (HR) stimulated by such biotic and abiotic effectors^39^. An oxidative burst induced by HR or another ROS-mediated defense response result in H_2_O_2_ buildup, which could explain the phenolic and lignin enhancement after biofertilizer application. The higher amount of lignin and the phenolic compounds reinforce the plant cell wall turning it into a physical barrier to pathogen penetration^40^.

Thus, both, milk-based products and biofertilizer are alternatives not only for organically but also conventionally produced kale for controlling black rot and promoting benefits to human health by nutritional enhancement and increasing the antioxidant activities.

## Methods

### Bacterial suspension culture

A virulent isolate of *X. campestris pv. campestris* isolated from symptomatic leaves was used. For experiments, *X. campestris pv. campestris* was grown in Nutrient Agar (NA) at 25 °C for 72 h. The bacterial cells were scraped from the NA plates, suspended in sterilized distilled water and the concentration adjusted to 5 × 10^6^ colony forming units mL^−1^ from optical density measurements (OD_600_ nm absorbance of 0.3 adjusted for 5 × 10^8^ UFC.mL^−1^)^41^. The inoculation was performed at the end of the day, eight days after plant treatment with each plant protectant, i.e. 23 days after transplanting.

### Plant material

Kale (*B. oleraceae* var. *acephala*) cv ‘Manteiga’ was used in all the experiments. The seedlings were obtained from a commercial seedling nursery (Casa da Semente, Lavras/Brazil). The seedlings were cultivated in 200-cell polystyrene trays for 35 days and transplanted to the field or greenhouse, depending on the specific experiment.

### Products details and plant spray

The same cow whey-milk and raw-milk (Laticínios Verde Campo, Lavras/Brazil) was used for all trials, inorganic ingredients for lime sulphur and Bordeaux mixtures were obtained from local market (Casa das Sementes, Lavras/Brazil) and cow manure from Milk-based cattle farm (Experimental station of Animal Science Department, Universidade Federal de Lavras, Lavras/Brazil). The milk-based products were immediately frozen (−20 °C) in individual vials and thawed overnight at room temperature before each use^14^. The biofertilizer was prepared according to Santos^42^. The cow’s manure (30 kg) was immersed in 60 L of water with one kilogram of sugarcane molasse, in a 200 L plastic barrel, homogenized and closed for an anaerobic fermentation for 30 d. The Bordeaux mixture (BM) [20 g/L CuSO_4_ + 20 g/L Ca(OH)_2_] and Lime Sulphur (LS) [250 g/L S + 125 g/L Ca(OH)_2_] were used according to Bettiol *et al*.^43^. The first spray was performed 35 days after transplanting and it was repeated 3 more times every two weeks.

### Trials schedule

For the non-inoculated experiment, plants were harvested and evaluated for plant yield, the centesimal composition, macro and micronutrient contents. The inoculated plant experiments encompassed the evaluation of black rot severity progress. The two treatments determined as most promising in the field trials for the disease control were further evaluated in greenhouse experiments for defense-related enzymes.

### Field trials

The experiments were carried out at the experimental station ‘Technological Development Campus’, Hortiagro located at 21°14′16′′S and 45°08′00′′W in a 500 m² field (20 m × 25 m per treatment) where vegetables are continuously produced in a rotation system for over 10 years. The rows were covered with white plastic mulch and drip irrigated when necessary to reach 70% field capacity. Fertilization was performed before transplanting by loading 20 g of a 08:24:14 (N:P:K) per plant. Weeding was performed when necessary with a hoe and pest control (*Plutella xylostella*) was performed by harvesting the older leaves and spraying *Bacillus thuringiensis* var. *kurstaki* (BT-TURBO MAX^TM^, Biovalens, Uberaba/Brazil) every two weeks when the first caterpillars were detected. Treatments were: i) Biofertilizer 20% v/v in water (BioF), ii) Raw-milk 10% v/v in water, iii) Whey 10% v/v in water, iv) Lime sulphur 0, 3 °Bê (Baumé degree), v) Bordeaux mixture 100% and vi) water control. The inoculated experiments were carried out after the non-inoculated ones to avoid cross-contamination. To assure plant infection, plants were overhead sprinkle-irrigated for 12 h continuously after inoculation. Plants were assessed for disease severity 5, 10 and 15 days after the inoculation with the diagrammatic scale of Nuñez *et al*., (2014)^44,45^. A total of four experiments were carried out using the same set of treatments, two of the experiments were not inoculated with any pathogen and two of them were inoculated with *X. campestris pv. campestris*. The non-inoculated ones were carried out prior to the inoculated ones.

### Yield and foliar nutrient content

Plants from both inoculated and non-inoculated experiments were evaluated for plant yield and nutrient content. At the last time point, leaves were harvested from the central four plants per plot and weighed to determine the leaf yield. Leaves were further evaluated for its nutrient content. From the harvested leaves, four of them per plot were used for nutrient analysis, always choosing the first fully expanded leaf from different plants within the plot. The samples were dried out in the oven at 70 °C, crushed in a mill, homogenized and weighed (0.5 g per sample). The sample was digested in a mix of nitric (4 mL) and perchloric (2 mL) acids. Later, the extract was diluted for nutrient determination by atomic absorption spectroscopy according to Malavolta^46^. Data were expressed as g.kg^−1^ and mg.kg^−1^ of dry weight for macronutrients (N, P, K, Ca, Mg, and S) and mg.kg^−1^ for micronutrients (B, Cu, Zn, Mn, and Fe) (Supplementary Tables 1–2).

### Centesimal composition, antioxidant activity (DPPH) and kale yield

From the same leaf sampling used to analyze the nutrient content, the centesimal composition (nutraceutical value) was also determined and encompassed humidity, ether extract, crude protein, crude fiber and ash fraction^47^. The humidity was determined according to the gravimetric technique, which was used in the heat ventilated oven at 65 °C until constant weight is obtained from kale. The ether extract (lipids) was determined by extraction with an organic solvent (ethyl ether), by the extractor, Crude protein determination was made through distillation nitrogen content in Microkjedahl apparatus (semi-micro), using the factor 6.25, carried the calculation of the crude protein content. The determination of crude fiber was performed by acid hydrolysis, by gravimetric method. Ash fraction was determined gravimetrically, evaluating the weight loss of the material subjected to heating in a muffle at 550 °–660 °C. The antioxidant activity was determined from the same leaf sampling, as well. The analysis was conducted based on diphenylpicrylhydrazyl (DPPH 60 µM) measurement (Rufino *et al*., 2007). Therewith, the scavenged DPPH was measured from 0 to 60 µM, in relation to the control treatment. The plant yield was measured by weighting the total amount of leaves at the last time point in grams from all the plants within the plot.

### Statistical analysis of field trials

The plants in fields were distributed in a randomized-block design with four replicates and 16 plants per plot. The experiment was performed twice for each condition. Data from different experiments were tested for normality by Shapiro Wilk test and submitted to one-way analysis of variance (ANOVA) followed by Tukey’s test (P = 0.05) in the plant protection against black rot, foliar nutrient and centesimal content assays. Yield and centesimal data were submitted to Hartley Equal Variance Test to check for homoscedasticity and, therefore the possibility of pooling data obtained from different experiments together. Repeated data sets were analyzed separately when not passing such test. Pearson’s correlation was determined between all evaluated variables. The increment and decrement data were estimated by percentage of increase or decrease calculation, comparing each treatment mean with the control mean, then the original Tukey test data are presented in Suplementary tables. All analyses were performed in the R software^48^.

### Defense-related enzyme trials

The greenhouse experiments were carried out at the Plant Pathology Department, Universidade Federal de Lavras. The area of the experiment was 150 m² (10 m × 13 m per treatment). The products were sprayed 35 days after transplant. Suspensions of i) Raw-milk; ii) Biofertilizer; iii) Control with water; iv) *X. campestris pv. campestris* were sprayed on the seedlings. The *X. campestris pv. campestris* was inoculated 5 days after spraying the products (120 h). The third pair of leaves of kale seedlings was used in this assay for enzyme activity quantification^49^. Control and treated leaves were sampled at 0, 24, 48, 96, 120, 144, 168 and 192 h after plant sprays. Those leaves were immediately frozen in liquid nitrogen, ca. 900 mg was ground to a fine powder and store at −80 °C until enzyme extraction.

### Assessment of defense enzymes

For the extraction of phenylalanine ammonia-lyase (PAL, EC 4.3.1.5), 700 μL of extraction buffer (50 mM of NaPO_4_ [pH 6.5], 1 mM PMSF-Phenylmethylsulfonyl fluoride) and 1% PVP- Polyvinylpyrrolidone) was added to 200 mg of each sample, mixed by inverting and immediately centrifuged at 14,000 rpm for 25 min at 4 °C. The supernatant was used for the enzyme activity assay by mixing 5 µL of enzymatic extract to 145 µL Tris-HCl [pH 8.8], 50 µL of 40 mM L-Phenylalanine (≥98%, Sigma-Aldrich), incubated for 20 min at 37 °C, absorbance was determined at 2 min intervals for 20 min at 280 nm and expressed as Δ280 nm mg.P^−1^.min^−1^ ^50^. To measure catalase (CAT, EC 1.11.1.6) activity, 10 µL of the supernatant was mixed with 100 µL of 200 mM of KH_2_PO_4_ [pH 7], 250 mM H_2_O_2_ and 80 µL distilled water, incubated for 3 min at 25 °C and absorbance reads were done at 0.5 min-intervals for 3 min. The CAT content was determined by measuring the rate of decrease in absorbance at 240 nm and the molar extinction coefficient of 18 mM^−1^ cm^−1^ ^51^. For peroxidase activity, 1300 μL the extraction buffer (400 mM of KH_2_PO_4_ [pH 7.8], 10 mM of Ethylenediaminetetraacetic acid-EDTA, 200 mM of ascorbic acid and distilled water) was added to 200 mg of each sample, mixed by inverting and immediately centrifuged at 14,000 rpm for 20 min at 4 °C, the supernatant was used for the subsequent enzyme. To measure guaiacol peroxidase (POX, EC 1.11.1.7) activity, 10 µL of the supernatant was mixed with 100 µL of 100 mM KH_2_PO_4_ [pH 7.0], 45 µL of 50 mM guaiacol (Sigma-Aldrich) and 45 µL of 125 mM H_2_O_2_, incubated for 10 min at 30 °C, absorbance was determined at 1 min intervals for 10 min at 480 nm and expressed as Δ480 nm mg.P^−1^.min^−1^^52^.

### Assessment of lignin and total phenol

The fourth pair of leaves of the plants as described in the assessment of defense enzymes (2.7) was used in these assays^49^. Samples were lyophilized for 76 h and ca. 30 mg of the lyophilized samples were transferred to 2-mL microcentrifuge tubes, homogenized with 1.5 mL of methanol 80%, shaken for 15 h at 150 rpm in the dark at room temperature and, thereafter, centrifuged at 12,000 rpm for 5 min at room temperature. The supernatant was used for total phenols and the precipitate was used for lignin quantification. For lignin quantification, the solid precipitated fraction was mixed with 1.5 mL of 80% methanol, shaken vigorously in a vortex, centrifuged at 12,000 rpm for 5 min, then the supernatant was discarded and the solid residue was dry for 48 h at room temperature. The samples were mixed with 1.5 mL of thioglycolic acid (0.15 mL) chloridric acid (HCl) 2 M (1.35 mL), mixed gently by inverting and placed in water at 90 °C for 4 h, centrifuged at 10,000 rpm for 10 min, the supernatant was discarded and the solid residue used. To the solid residue, 1.5 mL of distilled and deionized water was added, centrifuged at 10,000 rpm for 10 min, the supernatant discarded and the solid residue was resuspended with 1.5 mL 0.5 M sodium hydroxide (NaOH) and incubated in a shaker at 100 rpm for 15 h in the dark at room temperature. The samples were centrifuged at 10,000 rpm for 10 min, the supernatant was transferred to a new 2 mL microcentrifuge tube and mixed with 200 μL of 1 N HCl and incubated for 4 h at 4 °C, samples were again centrifuged at 10,000 rpm for 10 min, the supernatant was discarded and the solid residue resuspended in 2 mL 0.5 M NaOH. The lignin content was determined by reading the absorbance at 280 nm and expressed as μg of soluble lignin per milligram of dry mass^53^. For total phenol content, 150 μL of the methanol supernatant described above was mixed with 150 μL 0.25 N Folin-Ciocalteau for 5 min, homogenized with 150 μL 1 M Na_2_CO_3_ for 10 min and diluted with salt-free water for 1 h at room temperature. The total phenol content was determined by measuring absorbance at 725 nm and expressed as μg of chlorogenic acid per milligram of dry mass^54^.

### Statistical analyses of defence enzymes trials

For all assays, plants were distributed in a randomized-block design. For the products application assays we used 4 replicates per treatment with 4 plants per plot. The experiment was performed twice. Data from different experiments were tested for normality and submitted to one-way analysis of variance (ANOVA) followed by Tukey’s test (P = 0.05) for all analysis. Repeated data sets were analyzed separately. All analyses were performed in the R software^48^.

### Data availability

All data are available from the corresponding author upon reasonable request.

### Ethical statement

All methods and experiments were performed in accordance with relevant national and international guidelines and regulations. The present research do not require special approval by the ethics committee of Universidade Federal de Lavras, since the experiments to not encompass animal, human or GMOs (http://www.prp.ufla.br/comissoes-permanentes/ceua/).

## Electronic supplementary material

Supplementary Dataset 1

Supplementary Dataset 2

Supplementary Dataset 3

Supplementary Dataset 4
