# Supplementary material for: Bio-based products control black rot (Xanthomonas campestris pv. campestris) and increase the nutraceutical and antioxidant components in kale
Source: Sci Rep. 2018 Jul 5;8:10199. doi: 10.1038/s41598-018-28086-6 (PMC6033922; doi:10.1038/s41598-018-28086-6)
Supplement: Supplementary file 1 — Supplementary Dataset 1 [file 41598_2018_28086_MOESM1_ESM.docx]

**Bio-based products control black rot (***Xanthomonas campestris* pv. *campestris***) and increase the neutraceutical and antioxidant components in kale**

Andrés M.P. Nuñez, Gabriel A.A. Rodríguez, Fernando P. Monteiro, Amanda F. Faria, Julio C.P. Silva, Ana C. Monteiro, Carolina V. Carvalho, Luiz A.A. Gomes, Ricardo M. Souza, Jorge T. Souza, Flávio H.V. Medeiros.

**Table S1.** Contribution of bio-based products on the foliar mineral nutrition of kale (*Brassica oleraceae* var. *acephala*) at 23 days after transplanting

| Treatments | N | P | Ca | Mg | S | | Fe | Mn | | Cu |  |
| --- | --- | --- | --- | --- | --- | --- | --- | --- | --- | --- | --- |
|  | ----Means mg/Kg (ppm) ---- | | | |  | ---- Means dag/Kg (%) ---- | | | | | |
| Water | 2.570ab | 0.290c | 2.690a | 0.515b | 1.475a | | 81.00a | | 29.50b | 4.000a |  |
| Whey | 2.340bc | 0.285c | 2.370b | 0.505b | 1.645a | | 56.00b | | 25.00c | 3.500a |  |
| Lime | 2.495bc | 0.335b | 2.520a | 0.515b | 1.765a | | 59.50b | | 28.00b | 4.000a |  |
| Biofertilizer | 2.865a | 0.395a | 2.560a | 0.540a | 1.790a | | 77.00a | | 30.00b | 4.000a |  |
| Bordeaux mixture | 2.495bc | 0.320b | 2.035c | 0.420c | 1.700a | | 69.00b | | 28.00b | 2.350b |  |
| Raw milk | 2.205c | 0.245d | 2.580a | 0.555a | 1.475a | | 64.50b | | 39.00a | 4.000a |  |

Means followed by the same letter whitin the column are not significantly diferente according to Tukey’s test (P<0,05)
