# Supplementary material for: Bio-based products control black rot (Xanthomonas campestris pv. campestris) and increase the nutraceutical and antioxidant components in kale
Source: Sci Rep. 2018 Jul 5;8:10199. doi: 10.1038/s41598-018-28086-6 (PMC6033922; doi:10.1038/s41598-018-28086-6)
Supplement: Supplementary file 2 — Supplementary Dataset 2 [file 41598_2018_28086_MOESM2_ESM.docx]

**Bio-based products control black rot (***Xanthomonas campestris* pv. *campestris***) and increase the neutraceutical and antioxidant components in kale**

Andrés M.P. Nuñez, Gabriel A.A. Rodríguez, Fernando P. Monteiro, Amanda F. Faria, Julio C.P. Silva, Ana C. Monteiro, Carolina V. Carvalho, Luiz A.A. Gomes, Ricardo M. Souza, Jorge T. Souza, Flávio H.V. Medeiros.

**Table S2.** Contribution of different products on the foliar mineral nutrition of kale (*Brassica oleraceae* var. acephala) at 23 days after transplanting and eight days after inoculation with *Xanthomonas campestris* pv. *campestris*

| Treatments | N | P | Ca | Mg | S | | Fe | Mn | | Cu |  |
| --- | --- | --- | --- | --- | --- | --- | --- | --- | --- | --- | --- |
|  | ---- Means mg/Kg (ppm) ---- | | | |  | ---- Means dag/Kg (%) ---- | | | | | |
| Water | 4.530b | 0.465b | 1.485a | 0.345a | 1.450b | | 56.50a | | 37.50a | 3.500b |  |
| Whey | 4.725ab | 0.460b | 1.470a | 0.310b | 1.370c | | 70.00a | | 30.00c | 3.500b |  |
| Lime | 4.975a | 0.530a | 1.495a | 0.315b | 2.135a | | 57.00a | | 36.00a | 4.500a |  |
| Biofertilizer | 4.975a | 0.550a | 1.365b | 0.320a | 1.945a | | 60.00a | | 30.00c | 5.000a |  |
| Bordeaux mixture | 4.775ab | 0.490b | 1.565a | 0.355a | 1.670b | | 62.50a | | 33.00b | 3.000b |  |
| Raw milk | 5.005a | 0.460a | 1.270b | 0.295b | 1.370b | | 50.50a | | 23.50a | 3.000b |  |

Means followed by the same letter whitin the column are not significantly diferente according to Tukey’s test (P<0,05)
