# Supplementary material for: Bio-based products control black rot (Xanthomonas campestris pv. campestris) and increase the nutraceutical and antioxidant components in kale
Source: Sci Rep. 2018 Jul 5;8:10199. doi: 10.1038/s41598-018-28086-6 (PMC6033922; doi:10.1038/s41598-018-28086-6)
Supplement: Supplementary file 3 — Supplementary Dataset 3 [file 41598_2018_28086_MOESM3_ESM.docx]

**Bio-based products control black rot (***Xanthomonas campestris* pv. *campestris***) and increase the neutraceutical and antioxidant components in kale**

Andrés M.P. Nuñez, Gabriel A.A. Rodríguez, Fernando P. Monteiro, Amanda F. Faria, Julio C.P. Silva, Ana C. Monteiro, Carolina V. Carvalho, Luiz A.A. Gomes, Ricardo M. Souza, Jorge T. Souza, Flávio H.V. Medeiros.

**Table S3.** Pearson’s correlation between the contents of each analyzed nutrient and the black rot (*Xanthomonas campestris* pv. *campestris*) severity at 15 days after inoculation

| Nutrients | Experiment I | | Experiment II | |
| --- | --- | --- | --- | --- |
|  | Correlation(r) | P-value | Correlation(r) | P-value |
| N | -0,405 | 0,049* | -0,483 | 0,026* |
| P | -0,415 | 0,043* | -0,440 | 0,031* |
| S | 0,112 | 0,603 | 0,140 | 0,513 |
| Mn | -0,065 | 0,562 | -0,105 | 0,624 |
| B | 0,133 | 0,535 | 0,304 | 0,148 |
| Zn | -0,401 | 0,0519 | -0,339 | 0,010 |
| Cu | 0,003 | 0,989 | 0,461 | 0,023 |

. *Represents significance at 0.05 probability
