# Supplementary material for: Bio-based products control black rot (Xanthomonas campestris pv. campestris) and increase the nutraceutical and antioxidant components in kale
Source: Sci Rep. 2018 Jul 5;8:10199. doi: 10.1038/s41598-018-28086-6 (PMC6033922; doi:10.1038/s41598-018-28086-6)
Supplement: Supplementary file 4 — Supplementary Dataset 4 [file 41598_2018_28086_MOESM4_ESM.docx]

**Bio-based products control black rot (***Xanthomonas campestris* pv. *campestris***) and increase the neutraceutical and antioxidant components in kale**

Andrés M.P. Nuñez, Gabriel A.A. Rodríguez, Fernando P. Monteiro, Amanda F. Faria, Julio C.P. Silva, Ana C. Monteiro, Carolina V. Carvalho, Luiz A.A. Gomes, Ricardo M. Souza, Jorge T. Souza, Flávio H.V. Medeiros.

**Table S4.** Kale (*Brassica oleraceae* var. *acephala* cv. Manteiga) production and centesimal content with or without *Xanthomonas* *campestris* pv. *campestris* inoculation at 23days after transplanting or eight days after inoculation.

|  |  | | Yield | | | | | Lipid | Antioxidant | | | Crude protein | | | Crude fiber | | |
| --- | --- | --- | --- | --- | --- | --- | --- | --- | --- | --- | --- | --- | --- | --- | --- | --- | --- |
|  |  | | g | | | | | % | % | | | % | | | % | | |
| Without Bacteria | | | | | | | | | | | | | |  |  |  |  |
| Control | | |  | | 1014.25 a | | | 2.58d | | | 68.89 c | | | 4.88 b | | | 13.56 a |
| Whey | | |  | | 1052.25 a | | | 2.68d | | | 64.99 c | | | 4.69 b | | | 14.78 a |
| Lime Sulphur | | |  | | 966.25 a | | | 2.84c | | | 87.07 a | | | 4.86 b | | | 15.79 a |
| Biofertilizer | | |  | | 921.25 b | | | 3.48b | | | 65.56 c | | | 4.24 d | | | 16.01 a |
| Bordeaux mixture | | |  | | 881.75 b | | | 3.72a | | | 62.76 d | | | 5.06 a | | | 15.74 a |
| Raw milk | | |  | | 887.25 b | | | 3.30b | | | 84.08 b | | | 4.55 c | | | 14.01 a |
| With Bacteria | | | | | | | | | | | | | | | |  |  |
| Control | |  | | | | 555.75 c | 4.10c | | | 25.18 b | | | 8.80 d | | | 16.10 b | |
| Whey | |  | | | | 543.50 c | 4.72b | | | 33.07 a | | | 8.84 d | | | 17.10 b | |
| Lime Sulphur | |  | | | | 608.25 b | 3.82c | | | 23.28 b | | | 9.21 c | | | 20.30 a | |
| Biofertilizer | |  | | | | 687.75 a | 5.24a | | | 31.57 a | | | 9.39 b | | | 18.85 b | |
| Bordeaux mixture | |  | | | | 618.00 b | 4.89b | | | 29.73 a | | | 9.42 b | | | 23.05 a | |
| Raw milk | |  | | | | 625.50 b | 4.63b | | | 30.50 a | | | 9.75 a | | | 22.40 a | |

Mean values followed by the same letter within the columns are similar according to Scot’s Knot test at 5% probability.
